# The effect of facet joint injection on lumbar spinal stenosis with radiculopathy

**DOI:** 10.12669/pjms.344.15010

**Published:** 2018

**Authors:** Chan Jin Park, Young Duck Shin, Seung Woon Lim, Yoo Mee Bae

**Affiliations:** 1 Chan Jin Park, MD. Department of Anesthesiology and Pain Medicine, Chungbuk National University Hospital, College of Medicine, Chungbuk National University, Korea; 2 Prof. Dr. Young Duck Shin, MD, PhD. Department of Anesthesiology and Pain Medicine, Chungbuk National University Hospital, College of Medicine, Chungbuk National University, Korea; 3 Seung Woon Lim, MD, PhD. Department of Anesthesiology and Pain Medicine, Chungbuk National University Hospital, College of Medicine, Chungbuk National University, Korea; 4 Yoo Mee Bae, MD. Department of Anesthesiology and Pain Medicine, Chungbuk National University Hospital, College of Medicine, Chungbuk National University, Korea

**Keywords:** Facet joint, Spinal stenosis, Radiculopathy, Triamcinolone

## Abstract

**Objectives:**

Facet Joint Injection (FJI) is known to be effective in axial back pain, but the purpose of this study was to assess the effects of FJI on patients treated with it among those with Lumbar Spinal Stenosis (LSS).

**Methods:**

We conducted a retrospective database analysis and investigated electronic medical records of 125 LSS patients treated with FJI in the pain clinic of Chungbuk National University Hospital from November 2, 2016 to July 31, 2017. Sex, age, histories of low back surgery, complaining of neurogenic claudication, symptomatic sites of patients, FJI sites, number of sites of FJI, triamcinolone dosage, Numeric Rating Scale (NRS) before and after treatment, facet joint capsule rupture during treatment, and improvement of neurogenic claudication after treatment, were examined.

**Results:**

Among 125 patients, we investigated 91 patients who met the criteria. There was significant difference in NRS before and after treatment (p<0.000). Forty one patients with reduction of NRS more than 30% after FJI were allocated to effect group. FJI was more effective in patients who did not have the surgery (p=0.044), as well as those who showed an improved neurogenic claudication after treatment (p=0.001). Other measured values did not show statistical significances.

**Conclusions:**

FJI has relatively a lower risk and is simpler in terms of techniques than other interventional treatments performed within the spinal canal. Therefore, FJI may be another interventional treatment option in patients with pain by LSS. In the future, studies for FJI indication in LSS patients should be additionally required.

## INTRODUCTION

Lumbar Spinal Stenosis (LSS) is caused by an innate or acquired narrowing of the spinal canal. Clinical characteristics of LSS include pains such as leg pain while walking and lower extremity weakness, referred as to neurogenic claudication. Along with these symptoms, they also cover pain in leg segments applicable to the lumbar nerve root, as well as numbness, weakness, and loss of reflexes.^1^

Facet Joint Syndrome (FJS), a type of degenerative spondylosis, is one of the most common causes of Low Back Pain (LBP). Typically, FJS patients complain of referred pain in lower extremities. Specifically, lower lumbar FJS patients complain of referred pain in the hip and femoral region, and upper lumbar FJS patients in the flank, groin, and pelvic region.^2^ In physical examinations, tenderness around the facet joint, feeling heavy in the flank and sacral region, and sometimes cold symptoms may be reproduced in patients.^3^

Facet Joint Injection (FJI) is a procedure of injecting local anesthetics and steroids into facet joints for LBP by facet joint sprain or degenerative changes. It has relatively less side effects and is simpler in terms of techniques than intraspinal treatments due to its direct access to facet joints through paraspinal muscles.^4^ Studies reported that FJI is effective not only in axial back pain by facet joints but also lumbar spinal stenosis.^5^,^6^ Moreover, we showed previously an excellent outcome in patients diagnosed with LSS by performing FJI.^4^ Therefore, in this study, we tried to assess the effects of FJI on LSS patients based on retrospective analysis and literature review.

## METHODS

This study was approved by the Institutional Review Board (IRB) of Chungbuk National University Hospital (CBNUH; IRB No. H- 2017-07-018). Requirements such as written consent forms were exempted from the IRB. We conducted a retrospective database analysis and investigated Electronic Medical Records (EMRs) of 125 LSS patients treated with FJI in the pain clinic of our hospital from November 2nd 2016 to 31^st^ July 2017.

### Inclusion criteria

1) Patients complaining of characteristic symptoms of LSS such as radicular pain, radiculopathy, neurogenic claudication, and back pain; 2) Patients with radiological abnormal findings identical to symptomatic sites on Magnetic Resonance Imaging (MRI); 3) Patients confirmed to be accurately injected with contrast media to the joint space after checking FJI treatment image among the patients satisfying the conditions of 1) and 2). On the other hand,

### Exclusion criteria

1) Patients treated with another procedure in the same day of FJI treatment; 2) Patients treated in other departments in the same day of FJI treatment; 3) Patients newly administered with opioids from the day of FJI treatment to the evaluation day; 4) Patients not examined with MRI; 5) patients without revisit records within three months. Patients with addiction were not enrolled in this study.

### FJI

**Fig.1:**
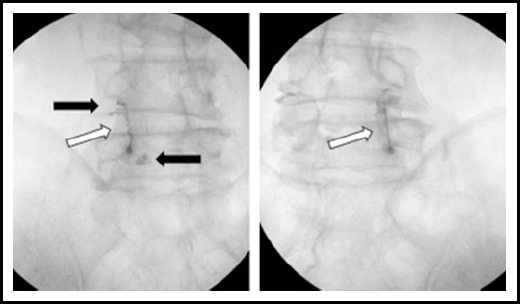
Facet joint injections at both L4/5 facet joints. This image shows that injected dye are contained in the facet joint space (white arrow) and leaks outside of joint (black arrow).

FJI was conducted by four specialists in the Department of Anesthesiology and Pain Medicine. Patients adopted a prone position and treatment sites were disinfected. Under C-arm fluoroscopy guidance, the specialists placed 80mm 25G Quincke spinal needles (Inter Kotra GmbH, Frankfurt am Main, Germany) into relevant facet joints and injected 0.2 - 0.3 ml contrast media into facet joints. After confirming that the contrast media were spread into the facet joint slit and the needles were accurately placed into the facet joints, they injected 1ml of 0.25% levobupivacaine mixed with triamcinolone into facet joint (Fig.1). At this time, a total of 20mg or 40mg triamcinolone was administered to patients treated with FJI in one site or two sites or more, respectively.

Steroids were unified by triamcinolone and the dose differences ranged from 20 mg to 40 mg depending on the number of procedures.

### Clinical Assessment

Based on medical examination records, medical progress notes, and procedure records in the EMRs of patients, sex, age, histories of low back surgery, complaining of neurogenic claudication, symptomatic sites of patients, FJI sites, number of sites of FJI, triamcinolone dosage, numeric rating scale (NRS; 0 - no pain, 10 - worst possible pain) before and after treatment, facet joint capsule rupture during treatment, and improvement of neurogenic claudication after treatment, were examined. These medical records were retrospectively investigated by one specialist in the Department of Anesthesiology and Pain Medicine.

### Statistical Analysis

We presented all measured values as mean ± standard deviation and number (%). Wilcoxon signed rank test was employed to analyze the difference of Numeric Rating Scale (NRS) before and after FJI. Patients who showed more than 30% decrease in NRS after, compared to before, FJI were assigned to effect group. Mann-Whitney test was used to compare age and NRS before FJI between effect and no effect groups. Chi-square test and Fisher’s exact test were performed to examine the relationship in sex, histories of low back surgery, complaints of neurogenic claudication, improvement of neurogenic claudication, symptomatic sites of patients, FJI sites, number of sites of FJI, and triamcinolone dosage between the two groups. In addition, the correlation between age of patients and decreased NRS after FJI was analyzed using Spearman’s rank correlation analysis. All data were statistically analyzed using Statistical Package for the Social Sciences (SPSS) version 23.0 (IBM Corp, Armonk, NY, USA). P values of less than 0.05 were considered to be statistically significant.

## RESULTS

Demographic and clinical data were analyzed in a total of 91 patients as shown in Table-I. FJI was averagely conducted 2.76 ±1.34 sites per patient. The number of levels for injection were 1.48 ±0.62 on average. Ten patients were treated with FJI in in only one site. In 90 patients, dyes spread outside facet joints during treatment. In all patients, there were no side effects such as lower extremity paralysis and infection. However, pain deteriorated in three patients.

**Table-I:** Demographics and clinical data.

Total No. of Pt.		91
Sex	Male	41(45.05%)
	Female	50(54.95%)
Age (Mean± SD)		67.45 ± 11.04
NRS (Mean± SD)		7.22 ± 1.56
Previous Op history	Yes	25(27.47%)
	No	66(72.53%)
Claudication	Yes	68(74.73%)
	No	23(25.27%)
Pain Site (Radiculopathy)	Both	43(47.25%)
	Rt	21(23.08%)
	Lt	27(29.67%)

No: number. Pt: patient. NRS: numeric rating scale. Op: operation. Rt: right. Lt: left.

Before and after treatment, the NRS of patients was 7.22 ±1.56 and 5.07 ±1.90, respectively. It decreased by 2.15 ±1.79. NRS was examined 3.10 ±1.99 weeks after treatment. As a result of Wilcoxon signed rank test, there was significant difference in NRS before and after treatment (p<0.000) (Table-II). However, there was no correlation between patient age and NRS change (p=0.093).

**Table-II:** Result of FJI.

			p-value
NRS*	Pre-FJI	7.22 ± 1.56	0.000
	Post-FJI	5.07 ± 1.91	
Effect**	Effect group	41(45.05%)	
	No effect group	50(54.95%)	

FJI: facet joint injection. NRS: numeric rating scale.

* Patients with NRS reduction of more than 30% after surgery were classified as effect group.

NRS decreased 30% or more in 41 patients (45.05%) after treatment. They were allocated to effect group. There was no difference in NRS between effect and no effect groups before treatment (p=0.068). This effect was not significantly related to sex (p=0.533), complaints of neurogenic claudication (p=0.860), number of sites of FJI (p=0.560), number of spinal levels of FJI (p=0.393), and triamcinolone dosage (p=0.750). However, it was significantly related to low back surgery before treatment. FJI was more effective in patients who did not have the surgery (p=0.044), as well as those who showed an improved neurogenic claudication after treatment (p=0.001) (Table-III).

**Table-III:** Possible clinical outcome predictors for facet joint injections at follow-up.

	Effect	No-effect	p-value
NRS at pre-FJI* (Mean± SD)	6.95 ± 1.70	7.44 ± 1.42	0.068
**Sex** **			0.533
Male	17	24	
Female	24	26	
Site**			0.170
Both	20	23	
Rt.	6	15	
Lt.	15	12	
Claudication**			0.860
Yes	31	37	
No	10	13	
Improve of Claudication**			0.001
Yes	28	20	
No	3	17	
Op History**			0.044
Yes	7	18	
No	34	32	
Level of FJI**			0.393
1 level	25	28	
2 level	12	20	
3 level	4	2	
No. of FJI**			0.560
1	5	5	
2	21	26	
3	1	0	
4	10	17	
6	4	2	
TA dose**			0.750
20mg	5	5	
40mg	36	45	

NRS: numeric rating scale. FJI: facet joint injection. Rt: right. Lt: left. Op: operation. TA: triamcinolone.* P value was less than 0.05

## DISCUSSION

LSS is a condition in which the spinal canal becomes narrow, thus pressing on nerves and blood vessels in it.^7^ This abnormal narrowing is caused by disc herniation, Ligamentum Flavum (LF) hypertrophy, facet joint capsule hypertrophy, and osteophyte formation.^4^ Symptoms of LSS include gluteal, lower extremity pain, LBP, weakness, and dysesthesia. In terms of walking motions, LSS causes neurogenic claudication. In particular, it improves by bending forward, sitting position, and recumbency. Degenerative LSS can be a nebulous diagnosis. The North American Spine Society (NASS) has recommended that patients should be diagnosed with degenerative LSS when their present illness and physical examination findings are in accordance with it and anatomic narrowing of the spinal canal or nerve root impingement are found through medical imaging examinations such as MRI, Computed Tomography (CT) myelography, and CT.^7^ FJS, one of the most common causes of LBP, is a pain attributed to structures that compose facet joints. These structures, including fibrous capsule, synovial membrane, hyaline cartilage, and bone, can cause FJS. Rarely, specific injuries can cause it as well. However, FJS is a degenerative disease mainly caused by repetitive stress and/or cumulative low-level trauma. Generally, patients complain of axial LBP. But they sometimes complain of referred pain in flank, hips, and legs. Moreover, if facet joints are badly inflamed, FJS can cause sciatica by stimulating adjacent spinal nerves. Specific physical examinations for FJS diagnosis are still unknown. CT, the best method to detect facet joint pathology, is strongly affected by age and is unrelated to patient symptoms as well.^8^ Accordingly, the most recommended diagnostic method is a diagnostic block, which includes medial branch block and intra-articular injection. It has been known that there is no difference between the two treatments in terms of accuracy.^9^ However, as these diagnostic blocks have a high false positive rate, the International Association for the Study of Pain (IASP) applies currently very rigid standards in conducting blocks to diagnose FJS.^10^

It is difficult to diagnose accurately spinal stenosis and FJS. Therefore, it is inappropriate to diagnose them only with the results of medical image examinations or diagnostic blocks. Patients with spinal stenosis or FJS should be diagnosed by considering their present illnesses and results of physical examinations in addition to those results.

Hwang SY et al. reported a retrospective study for FJI effects on LSS patients at risk of hemorrhage. FJI was effective in 25 (59.5%) out of 42 patients. On MRI, it was more effective in patients with mild-to-moderate central canal stenosis. In this study, it was assumed that medicines can be injected into the epidural space through facet joints. The authors injected 1ml medicines into each joint and additionally 2-4ml contrast media or 0.9% normal saline to induce the rupture of the facet joint capsule and the drug efflux into the epidural space. However, there was no correlation between discharge of contrast media and treatment effect.^5^ In addition, E. Shim et al. investigated which treatments were chosen as the third one by patients treated with both FJI and Epidural Steroid Injection (ESI) on different days within two months among those with lumbar central canal stenosis. As a result, 33 (66%) out of 50 patients chose FJI as the third treatment. Thirteen (68.4%) out of not-improved 19 patients treated with ESI as the first treatment improved after treated with FJI as the second treatment. Seven (53.8%) out of 13 patients treated with FJI as the first treatment improved. Accordingly, they demonstrated that FJI can be performed as an alternative treatment in patients with lumbar central canal stenosis.^6^

The possible mechanisms of FJI on LSS patients are assumed as follows: 1) A decompression effect of steroids injected into facet with hypertrophied facet joint. The authors conducted directly RF lesioning to the facet joint capsule, which reduced the size of hypertrophied facet joint capsule. Jo et al. reported a case that Radio Frequency (RF) lesioning was done in spinal stenosis patients nerves were decompressed.^11^ Accordingly, facet joint capsule edema, LF edema, or inflammatory fluid in the facet joint caused by facet joint degeneration were reduced by steroids injected into the facet joint, thus showing a decompression-like effect. Consequently, the symptoms improved; 2) A spreading effect of steroids and medicines injected into the facet joint directly to the epidural space. LF covers the anterior capsule of facet joints.^12^,^13^ However, lateral LFs are gradually thinned and are removed merging with the capsule.^14^ With degenerative changes, the capsule is consistently smaller and thinned. If medicines more than the joint capacity in the facet joint are administered, the weakened capsule is ruptured^15^,^16^ and the medicines can spread to the epidural space or around the intervertebral foramen.^17^ In addition, the medicines can spread by the cervical and lumbar facet joint and the communication of the retrodural space.^18^,^19^ Accordingly, medicines injected into the facet joint spread to the epidural space or around spinal nerves and they may take effect. This mechanism is almost similar to the hypothesis of Hwang ST et al. It is highly possible in that, in the present study, contrast media were discharged out of the facet joint in 98.9% of patients as well.

The present study allocated patients whose NRS decreased 30% after, compared to before, treatment to effect group. Forty one (45.05%) out of 91 patients were classified into effect group. Characteristics of effect group, including age, sex, pain severity before treatment, pain sites, number of sites of treatment, levels for treatment, and triamcinolone dosage, were not related to FJI effect. On the other hand, medical histories of back surgery and improvement of neurogenic claudication were related to this effect. It can be expected that medicines discharged out of the facet joint failed to reach properly pain-generating sites and showed a reduced effect on pain relief due to sequelae such as epidural adhesion after back surgery.^20^ However, Hwang SY et al. revealed that there was no correlation between the degree of spreading to the epidural space and the FJI effect.^5^ Furthermore, many studies have also reported that the severity of spinal stenosis or facet arthropathy were not related to ESI or FJI effect.^21^,^22^ As such further studies for why these treatments are more effective in non-operated patients should be conducted. Neurogenic claudication, a characteristic symptom of spinal stenosis, limits daily lives of patients or their motions and has a great effect on their quality of life.^23^

Thus, it is considered that FJI was more effective in patients with improved neurogenic claudication because patients felt greater satisfaction with their improved neurogenic claudication. Based on this, FJI effect cannot be attributed to the relief of facet joint pain accompanied in LSS patients.

Interventional treatments conducted within the spinal canal such as ESI have a number of serious side effects, including hypotension, spinal cord injury, spinal nerve injury, spinal cord infarction, paralysis, epidural hematoma, epidural abscess by infection, and Cerebrospinal Fluid (CSF) leakage and spinal headache by dural puncture. However, FJI can directly reach the facet joint if the needle passes only through paraspinal muscles and the facet joint capsule, thereby it is less invasive and has less side effects than treatments performed in the spinal canal such as local hemorrhage and oozing, local hematoma, nerve root stimulation. Hwang SY et al. indicated that FJI was safely conducted even in patients at risk of hemorrhage.^5^ In addition, it has a strong point that it is technically simple compared to other treatments performed within the spinal canal.^4^ However, a number of studies found that there are connected passages between the facet joint and the epidural space.^18^,^19^ A study reported that they are accessible to each other.^24^ Therefore, we should pay close attention to FJI like other treatments.

### Limitations of the study

1) There was a difference in the time interval when changes in NRS were assessed after FJI; 2) Periods of sustained FJI effect were not investigated; 3) Changes in NRS by administered oral medicines were not correctly reflected; 4) It is impossible to confirm which symptoms improved in the measurement of FJI effects; 5) This study investigated only improvement of claudication as a FJI effect and could not confirm the level of improvement in detail.

In conclusion, the new finding of this study is based on the hypothesis that steroids and drugs administered in facet joints are effective in spreading to the epidural space in patients with spinal stenosis. FJI has relatively a lower risk and is simpler in terms of techniques than other interventional treatments performed within the spinal canal. Therefore, FJI may be another interventional treatment option in patients with pain by LSS. Henceforth, future studies should be required concerning FJI indication in LSS patients.

### Authors Contributions

**YD:** Conceived, designed and editing of manuscript.

**SW, YM:** Manuscript writing.

**CJ:** Manuscript writing and revision of final draft.

**YD:** Takes the responsibility and is accountable for all aspects of the work in ensuring that questions related to the accuracy or integrity of any part of the work are appropriately investigated and resolved.

## References

[1] Genevay S, Atlas SJ (2010). Lumbar spinal stenosis. Best practice Res Clini Rheumato.

[2] Mooney V, Robertson J (1976). The facet syndrome. Clini Orthopaedics Related Res.

[3] Kim KH (2008). Spinal Joint Pain Syndrome. Korean J Pain.

[4] Jeon YW, Bae YM, Shin YD, Park SH, Choi JH, KH Y (2016). Would Facet Joint Steroid Injection Be Feasible Treatment in Spinal Stenosis?- Cases Report and Review of Literature. Int J Pain.

[5] Hwang SY, Lee JW, Lee GY, Kang HS (2013). Lumbar facet joint injection:feasibility as an alternative method in high-risk patients. Eur Radiol.

[6] Shim E, Lee JW, Lee E, Im T, Kang Y, Ahn JM (2017). Facet joint injection versus epidural steroid injection for lumbar spinal stenosis:intra-individual study. Clini Radiol.

[7] Kreiner DS, Shaffer WO, Baisden JL, Gilbert TJ, Summers JT, Toton JF (2013). An evidence-based clinical guideline for the diagnosis and treatment of degenerative lumbar spinal stenosis (update). The spine journal:Official J North Ame Spine Soci.

[8] Schwarzer AC, Wang SC, O'Driscoll D, Harrington T, Bogduk N, Laurent R (1995). The ability of computed tomography to identify a painful zygapophysial joint in patients with chronic low back pain. Spine.

[9] van Kleef M, Vanelderen P, Cohen SP, Lataster A, Van Zundert J, Mekhail N (2010). Pain originating from the lumbar facet joints. Pain practice:Official J World Ins Pain.

[10] Treede RD, Rief W, Barke A, Aziz Q, Bennett MI, Benoliel R (2015). A classification of chronic pain for ICD-11. Pain.

[11] Oh J, Jo D, Kim K, Shim J, Roh M (2016). Facetoplasty Using Radiofrequency Thermocoagulation for Facet Joint Hypertrophy. Pain physician.

[12] Destouet JM, Gilula LA, Murphy WA, Monsees B (1982). Lumbar facet joint injection:indication, technique, clinical correlation, and preliminary results. Radiol.

[13] Xu GL, Haughton VM, Carrera GF (1990). Lumbar facet joint capsule:appearance at MR imaging and CT. Radiol.

[14] Losiniecki AJ, Serrone JC, Keller JT, Bohinski RJ (2013). Lumbar ligamentum flavum:spatial relationships to surrounding anatomical structures and technical description of en bloc resection. J Neurolo Surg Part A, Cent Euro Neurosurg.

[15] Dreyfuss PH, Dreyer SJ, Herring SA (1995). Lumbar zygapophysial (facet) joint injections. Spine.

[16] Kalichman L, Hunter DJ (2007). Lumbar facet joint osteoarthritis:a review. Seminars in arthritis and rheumatism.

[17] Raymond J, Dumas JM (1984). Intraarticular facet block:diagnostic test or therapeutic procedure?. Radiol.

[18] Okada K (1981). Studies on the cervical facet joints using arthrography of the cervical facet joint. Nihon Seikeigeka Gakkai Zasshi.

[19] Murthy NS, Maus TP, Aprill C (2011). The retrodural space of Okada. Am J Roentgeno.

[20] Hu MH, Yang KC, Sun YH, Chen YC, Yang SH, Lin FH (2017). In situ forming oxidised hyaluronic acid/adipic acid dihydrazide hydrogel for prevention of epidural fibrosis after laminectomy. Euro cells Materi.

[21] Campbell MJ, Carreon LY, Glassman SD, McGinnis MD, Elmlinger BS (2007). Correlation of spinal canal dimensions to efficacy of epidural steroid injection in spinal stenosis. J spinal disorders techniques.

[22] Hechelhammer L, Pfirrmann CW, Zanetti M, Hodler J, Boos N, Schmid MR (2007). Imaging findings predicting the outcome of cervical facet joint blocks. Euro Radiol.

[23] Drury T, Ames SE, Costi K, Beynnon B, Hall J (2009). Degenerative spondylolisthesis in patients with neurogenic claudication effects functional performance and self-reported quality of life. Spine.

[24] Yu HJ, Park CJ, Yim KH (2016). Successful Treatment of a Symptomatic Discal Cyst by Percutaneous C-arm Guided Aspiration. Korean J Pain.

